# Basic perceptual changes that alter meaning and neural correlates of recognition memory

**DOI:** 10.3389/fnhum.2015.00049

**Published:** 2015-02-11

**Authors:** Chuanji Gao, Molly S. Hermiller, Joel L. Voss, Chunyan Guo

**Affiliations:** ^1^Beijing Key Laboratory of Learning and Cognition, Department of Psychology, College of Education, Capital Normal UniversityBeijing, PR China; ^2^Department of Medical Social Sciences, Ken & Ruth Davee Department of Neurology, and Interdepartmental Neuroscience Program, Feinberg School of Medicine, Northwestern UniversityChicago, IL, USA

**Keywords:** color change, perceptual processing, conceptual processing, recognition, meaning

## Abstract

It is difficult to pinpoint the border between perceptual and conceptual processing, despite their treatment as distinct entities in many studies of recognition memory. For instance, alteration of simple perceptual characteristics of a stimulus can radically change meaning, such as the color of bread changing from white to green. We sought to better understand the role of perceptual and conceptual processing in memory by identifying the effects of changing a basic perceptual feature (color) on behavioral and neural correlates of memory in circumstances when this change would be expected to either change the meaning of a stimulus or to have no effect on meaning (i.e., to influence conceptual processing or not). Abstract visual shapes (“squiggles”) were colorized during study and presented during test in either the same color or a different color. Those squiggles that subjects found to resemble meaningful objects supported behavioral measures of conceptual priming, whereas meaningless squiggles did not. Further, changing color from study to test had a selective effect on behavioral correlates of priming for meaningful squiggles, indicating that color change altered conceptual processing. During a recognition memory test, color change altered event-related brain potential (ERP) correlates of memory for meaningful squiggles but not for meaningless squiggles. Specifically, color change reduced the amplitude of frontally distributed N400 potentials (FN400), implying that these potentials indicated conceptual processing during recognition memory that was sensitive to color change. In contrast, color change had no effect on FN400 correlates of recognition for meaningless squiggles, which were overall smaller in amplitude than for meaningful squiggles (further indicating that these potentials signal conceptual processing during recognition). Thus, merely changing the color of abstract visual shapes can alter their meaning, changing behavioral and neural correlates of memory. These findings are relevant to understanding similarities and distinctions between perceptual and conceptual processing as well as the functional interpretation of neural correlates of recognition memory.

## Introduction

*I do not like green eggs and ham! I do not like them, Sam-I-am*.

*-Theodore Seuss Geisel, 1960*

Although perceptual processing and conceptual processing are often treated as distinct entities, it is difficult to precisely identify their differences. For example, changing simple perceptual characteristics of a visual stimulus (such as color, size, and orientation) is generally thought to change perceptual but not conceptual processing (Jolicoeur, 1985; Masson, 1986; Jacoby and Hayman, 1987; Roediger and Blaxton, 1987; Graf and Ryan, 1990; Biederman and Cooper, 1992; Groh-Bordin et al., 2006; Uttl et al., 2006; Ecker et al., 2007a,b). However, there are many instances when slight changes to perceptual features greatly change the meaning of a stimulus. For example, imagine purchasing a loaf of white bread, but when you get home you discover that it is green. Would you still eat it? Likewise, the meaning of a green vs. a red light is quite different while in traffic. Thus, it is difficult to categorize perceptual vs. conceptual processing (Martin, 2007; Schendan and Maher, 2009). In the current experiment, we sought to better understand the nature of relevant distinctions by manipulating a simple perceptual feature (color) in conditions when this perceptual change would, vs. would not, also produce a change in meaning.

The distinctions between perceptual and conceptual processing are highly relevant to better understanding of the neural correlates of recognition memory. The process of making judgments about prior encounters is defined as recognition memory, which can be subdivided into two expressions termed recollection and familiarity. Recollection involves the retrieval of specific details about something recognized (such as when and where an event took place), whereas familiarity refers to simply knowing that something previously occurred or was experienced without recalling specific details (Mandler, 1980; Yonelinas, 2002). Previous event-related brain potential (ERP) studies have attempted to identify distinct neural correlates of recollection and familiarity. These studies have often found that recollection is associated with a 500–700 ms parietal effect termed the late-positive complex (LPC) whereas familiarity is associated with a 300–500 ms negative-going frontal effect termed the frontal N400 (FN400) (see Mecklinger, 2006; Rugg and Curran, 2007 for review). However, neural measures collected during a recognition memory test are sensitive not only to explicit memory processes such as recollection and familiarity, but also to various expressions of implicit memory. Many findings indicate that FN400 effects could index conceptual implicit memory instead of familiarity (for review, see Paller et al., 2007; Dew and Cabeza, 2011; Voss et al., 2012b). Indeed, some findings suggest that implicit perceptual and conceptual processing not only occurs during recognition memory testing, but can sometimes influence recognition, particularly familiarity-based recognition (Whittlesea et al., 1990; Jacoby, 1991; Wagner and Gabrieli, 1998; Voss and Paller, 2006; Lucas et al., 2012). The general logic of our study, as described below, is that manipulations of conceptual processing via color change would influence neural correlates of recognition memory to the extent that implicit conceptual processing occurred during recognition memory testing.

Several studies manipulated color to test for perceptual contribution to familiarity and concluded that perceptual processing can modulate familiarity because color change had an influence on neural measurements of familiarity (Groh-Bordin et al., 2006; Ecker et al., 2007a,b). However, it is unclear based on this evidence whether perceptual vs. conceptual factors were at play. That is, did color change modulate perceptual processing related to familiarity, conceptual processing related to familiarity, or some combination of perceptual and conceptual processing? Furthermore, because the neural correlates of familiarity are not fully specified and likely not absolute across all testing situations (Bridger et al., 2012; Paller et al., 2012), it is difficult to know based on neural measures alone whether perceptual or conceptual changes are influencing familiarity vs. conceptual processing (Voss et al., 2012b).

In the current experiments, we aimed to verify the effects of conceptual implicit memory on neural correlates of recognition memory and the effect of color change on these influences. Color-change manipulations were used with stimuli that vary in meaningfulness in an attempt to separate processing that is relatively perceptual (color change for meaningless stimuli) from processing that is relatively conceptual (color change for meaningful stimuli), measured during recognition memory testing. “Squiggles” were used as stimuli because they vary widely and idiosyncratically in perceived meaningfulness (Voss and Paller, 2007; Voss et al., 2010b, 2012a). In the current experiments, squiggles were separated into meaningful and meaningless groups based on individualized ratings. We examined how the same manipulation (color change from study to test) produces different effects depending on whether the squiggles were meaningful or meaningless. This allowed us to identify neural correlates of perceptual and conceptual processing during recognition memory testing for the same stimulus set and task.

**Figure 1:**
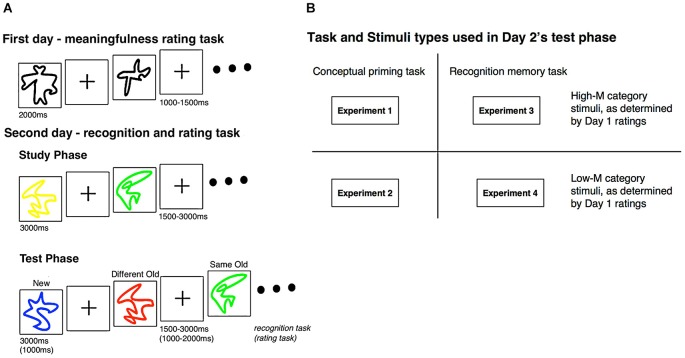
**Experimental design. (A)** During the first day of the experiment, subjects categorized squiggle stimuli as High-M or Low-M using a 4-point meaningfulness rating scale (see text). Subjects returned for a second day of testing, during which study-test blocks were presented. Subjects studied squiggles presented in a uniform color. At test, the same squiggles were presented in either the same color as for study (same-old) or in a different color (different-old). These squiggles were intermixed with new squiggles. **(B)** For Experiments 1 and 2, a conceptual priming test was used (speeded meaningfulness rating). For Experiments 3 and 4, a recognition memory test was used (modified remember/know procedure). The stimuli used in Day 2 was the same stimuli that was established as either High-M or Low-M during the Day 1 rating task; Experiments 1 and 3 used High-M squiggles while Experiments 2 and 4 used Low-M squiggles.

A general overview of the experiment design is provided in Figure 1. In Experiments 1 and 2, we aimed to determine effects of color change on behavioral measures of conceptual priming in order to test the hypothesis that conceptual implicit memory occurs selectively for meaningful stimuli and that color change therefore only influences behavioral measures of priming for meaningful stimuli. In Experiment 1, we performed conceptual priming tests with color change manipulations for stimuli that subjects found to be relatively high in meaning (High-M) whereas in Experiment 2 we performed the same tests for stimuli that subjects found to be relatively low in meaning (Low-M). Thus, we predicted that behavioral correlates of conceptual priming and of the color-change manipulation would be found specifically in Experiment 1, not in Experiment 2. In Experiments 3 and 4, ERPs were used to identify the effects of color change on neural correlates of recognition memory for meaningful and meaningless squiggles, in order to isolate neural signals of implicit conceptual processing operative during recognition memory testing. In Experiment 3, recognition memory tests were given for stimuli that subjects found to be relatively High-M, whereas in Experiment 4 we performed the same tests for stimuli that subjects found to be relatively Low-M. We hypothesized that the color-change manipulation would influence ERP correlates of recognition memory only for stimuli in Experiment 3, not for stimuli in Experiment 4. By comparing results across experiments, we were able to test whether the effects of color change from study to test on the neural correlates of recognition memory are selective for High-M stimuli (Experiment 3 vs. Experiment 4), as likely due to the fact that High-M stimuli selectively support conceptual priming and influences of color change on conceptual priming (Experiment 1 vs. Experiment 2). Furthermore, we anticipated that differences in meaningfulness and in the effects of color-change manipulation would be selective for FN400 ERPs, as we predict that these ERPs reflect conceptual implicit memory during recognition memory testing (Voss et al., 2012b).

## Materials and methods

### Experiments 1 and 2: effects of color change on conceptual priming for meaningful vs. meaningless stimuli

#### Subjects

Fourteen students (12 females, 19–26 years of age, right-handed) from Capital Normal University participated in Experiment 1, and another fourteen students (12 females, 19–27 year of age, right-handed) also from Capital Normal University participated in Experiment 2. All participants had normal or corrected-normal vision and did not have color blindness. They were paid for their participation. The Capital Normal University’s Institutional Review Board approved this study.

#### Materials

Four hundred and ninety five squiggles were used as stimuli (Figure 1). 316 squiggles were taken from two previous studies (Groh-Bordin et al., 2006; Voss and Paller, 2007) and the remaining were created via hand-deformation of squares, circles, and triangles. In accordance with prior studies (e.g., Voss and Paller, 2007; Voss et al., 2010b), in our current two experiments, results of first-day meaningfulness ratings indicated that squiggles varied widely and idiosyncratically in how individuals perceived their meaningfulness, which justifies the need to assess meaningfulness on an individual basis. Thus meaningful (High-M) or meaningless (Low-M) stimuli in second-day experiments were customized to each subject. Squiggles were presented on a 15-inch computer cathode ray tube (CRT) monitor on a white background within four degrees of the visual angle. A cross fixation was presented in the center of the screen during each inter-stimuli interval (ISI).

#### Procedure

Experiments 1 and 2 were each conducted over two separate days. On the first day, subjects were presented with 11 blocks of black squiggles (45 squiggles in each block). The squiggles were shown in the middle of the screen for 2000 ms with a variable 1000–1500 ms ISI. The order the squiggle were presented in each block was auto-randomized, and the order of the blocks was pseudo-randomized. Subjects were instructed to rate the meaningfulness of each squiggle on a scale of 1 (meaningful) to 4 (meaningless). A response of “1” indicated the squiggle looked like a “nameable object, face, or animal,” “2” indicated “a more abstract nameable object, face, or name,” “3” represented a squiggle that “does not look like anything nameable, but is in some way meaningful,” and “4” if it was a “random collection of lines that is in no way meaningful.” Later, responses were coded as either High-M (scores 1 and 2) or Low-M (scores 3 and 4).

Subjects returned 1–3 days later for the second part of the study, which consisted of five study-test blocks. For Experiment 1, the stimuli that were used for the Day 2 study-test blocks were all from the High-M category based on Day 1 ratings. For Experiment 2, all stimuli during Day 2 were from the Low-M category based on Day 1 ratings.

For both experiments, each study-test block consisted of one study phase and one test phase. During the study phases, subjects were presented four blocks of colored (red, yellow, green, or blue) squiggles (24 squiggles in each block) that had been viewed during Day 1. Each squiggle appeared for 3000 ms with a variable 1500–3000 ms ISI. The test phase began nearly 45 s after the study phase completed, during which time subjects counted backwards by threes from a specified number. During the test phase, squiggles were presented individually for 1000 ms with a variable 1000–2000 ms ISI. Forty squiggles were presented in each test phase: 24 which had been shown during the previous study phase (12 squiggles of identical color, 12 squiggles of different color) and 16 squiggles which were not presented during the study phase. Squiggles of same and different colors, as well as the transitions, were counterbalanced for each individual. Subjects were instructed to rate the meaningfulness of each squiggle on a scale of 1 (meaningful) to 4 (meaningless) during the test, but response speed was emphasized. Subjects were told that although they may have already seen some of the squiggles, ratings should be made as fast as possible and irrespective of previous experience and ratings, as trying to remember such information could delay response speeds (Figure 1).

### Experiments 3 and 4: ERP correlates of the influence of color change on recognition memory for meaningful vs. meaningless stimuli

#### Subjects

Fourteen students (10 females, 19–25 years of age, right-handed) from Capital Normal University participated in Experiment 3, and 16 students (9 females, 19–27 years of age, right-handed) also from Capital Normal University participated in Experiment 4. All participants had normal or corrected-normal vision and did not have color blindness. They were paid for their participation. The Capital Normal University’s Institutional Review Board approved this study.

#### Materials

One thousand eight hundred minimalist squiggles were used as stimuli. 316 squiggles were taken from two previous studies (Groh-Bordin et al., 2006; Voss and Paller, 2007) and the remaining were created via hand-deformation of squares, circles, and triangles. In accordance with Experiments 1 and 2, there was a lack of agreement among participants regarding meaningfulness in first-day meaningfulness ratings. Thus High-M or Low-M stimuli in second-day experiments were customized to each participant, as in Experiments 1 and 2. Squiggles were presented on a 15-inch computer CRT monitor on a white background within about four degrees of visual angle. A fixation cross was presented in the center of the screen during each ISI.

#### Procedure

As was the case for Experiments 1 and 2, Experiments 3 and 4 were both conducted over two days. On the first day, subjects were presented with 10 blocks of black squiggles (180 squiggles in each block). The squiggles were shown in the middle of the screen for 2000 ms with a variable 1000–1500 ms ISI. The order of squiggles presented in each block was auto-randomized, and the order of blocks was pseudo-randomized. Meaningfulness ratings were made and High-M and Low-M categories formed, as in Experiments 1 and 2.

Subjects returned 1–3 days later for Day 2, which consisted of 20 study-test blocks. Each study-test block consisted of a study phase followed by a test phase. During the study phase, subjects were presented with 28 colored (red, yellow, green, or blue) squiggles. Each squiggle appeared for 3000 ms with a variable 1500–3000 ms ISI with two filler pictures at the beginning and end of the block that were not later tested (to avoid primacy and recency effects). Subjects were instructed to memorize each item and its color. The test phase began nearly 45 s after the study phase, and subjects counted backwards by threes from a specified number during the break. During the test phase, squiggles were presented for 3000 ms each with a variable 1500–2000 ms ISI. Forty squiggles were presented in each test phase: 24 that had been shown during the study phase (12 squiggles of identical color, 12 squiggles of different color) and 16 squiggles that were not presented during the study phase. Squiggles of same and different colors, as well as the transitions, were counterbalanced for each individual. Subjects were instructed to categorize each squiggle using one of four responses: (1) “remember,” indicating recollection of the stimulus, including color or another detail from the study phase; (2) “know,” indicating familiarity for the squiggle from the study phase, but no recollection of color or other details; (3) “guess,” indicating the inability to determine if the squiggle was presented during the study phase or not; or (4) “new,” indicating confidence that the squiggle was not presented during the study phase (Figure 1).

Experiments 3 and 4 followed the same methodology except Experiment 3 used squiggles on Day 2 that had been rated as High-M on Day 1, and Experiment 4 used squiggles on Day 2 that had been rated as Low-M on Day 1 (as was the case for Experiments 1 and 2, respectively).

#### ERP methods

Continuous electroencephalographic (EEG) recordings were measured from 62 scalp sites with a NeuroScan SynAmps system (NeuroScan Inc. Sterling, Virginia, USA) during Day 2 study and test phases. One subject was excluded from ERP analyses because of excessive EEG artifacts. In accordance with the extended international 10–20 systems (Picton et al., 2000), 62 scalp sites were targets with Ag/AgCl electrodes embedded in an elastic cap. Left mastoid was used as a reference site on-line. Signals were re-referenced offline to averaged mastoids. Four additional channels were used for monitoring horizontal and vertical eye movements (horizontal electrooculogram were recorded bipolarly from electrodes placed 1 cm to the left and right of the outer canthi; vertical electrooculogram were recorded bipolarly from electrodes placed above and below the left eye). Sampling rate was 500 Hz with a bandpass of 0.05–40 Hz. Impedance was less than 5 kΩ. Each epoch began 200 ms prior to stimulus onset and lasted 1400 ms. Baseline corrections were performed using mean amplitudes of pre-stimulus onset. Trials exceeding ±75 µv were rejected. EOG blink artifacts were corrected statistically using a linear regression estimate (Semlitsch et al., 1986).

ERP amplitudes were averaged over three sets of midline electrodes along the anterior-posterior axis (frontal: F3, Fz, F4; central: C3, Cz, C4; parietal: P3, Pz, P4). Analyses focused on the conditions in which subjects made correct responses. On average, the minimum trials per condition were 15. Conditions were made based on study-test consistency and old/new status (same-old, different-old, and new) as well as on the four recognition response types (remember, know, guess, and new), separately for High-M squiggles in Experiment 3 and Low-M squiggles in Experiment 4.

Latency intervals (350–500 ms, 500–700 ms) were selected based on review of the waveforms and on existing literature regarding FN400 effects and LPC effects, as were electrode clusters (Rugg and Curran, 2007). Statistical analyses of ERP waveforms focused on amplitude values averaged over latency intervals (350–500 ms, 500–700 ms) and over electrode clusters (frontal, central, parietal). Peak latency of FN400 effects for the same-know condition and the different-know condition were calculated by measuring the time latency of minimum peak amplitude between 250–500 ms. Waveforms were smoothed with a 20 Hz low-pass-zero phase-shift Butterworth filter for presentation purposes only.

In our study, all analysis used the Greenhouse-Geisser correction for non-sphericity when necessary, and Greenhouse-Geisser corrected degrees of freedom are presented in the text. Bonferroni-corrected data are presented for *post-hoc* pairwise comparisons.

## Results

### Experiments 1 and 2

In Experiment 1, the average probability that a squiggle was rated as High-M was 43.9% (SE = 3.2%) and as Low-M was 55.4% (SE = 3.1%). In Experiment 2, the average probability that a squiggle was rated as High-M was 36.5% (SE = 2.8%) and as Low-M was 63% (SE = 2.8%). The consistency of meaningful ratings between Day 1 and Day 2 were calculated. In Experiment 1, High-M had an overall consistency of 49.1% (SE = 2.2%). Old items rated as High-M had a consistency of 52.2% (SE = 2.7%) while new items rated as High-M had 44.5% (SE = 2.2%). In Experiment 2, Low-M had an overall consistency of 60.8% (SE = 3.7%), where old items were 56.6% (SE = 4.0%) and new items were 67.1% (SE = 3.5%).

Response times (RTs) were collected for each meaningful rating and used to measure the difference between High-M and Low-M RTs. Kolmogorov-Smirnov tests of normality of distribution were calculated for all Experiment 1 and 2 RTs, which reveal that the RTs do not significantly deviate from normal (all *p* values were > 0.1). In Experiment 1, the average RT of Day 1 ratings for High-M was 1467 ms (SE = 91.4) and for Low-M was 1471 ms (SE = 97.7). Results of a *t*-test [*t*_(13)_ = −0.137, *p* = 0.893] revealed no significant difference between the High-M and Low-M RTs. In Experiment 2, the average RT of Day 1 ratings for High-M was 1481 ms (SE = 68) and for Low-M was 1446 ms (SE = 77.6). Again, a *t-test* [*t*_(13)_ = 1.406, *p* = 0.183] showed no significant difference between the RTs.

To measure conceptual priming, the differences in RTs for old vs. new squiggles during Day 2 were used. In Experiment 1, RTs for old High-M squiggles were 30 ms faster than for new squiggles [*t*_(13)_ = 5.736, *p* < 0.001]. In Experiment 2, RTs for Low-M squiggles were not significantly different from RTs for new squiggles [*t*_(13)_ = 0.636, *p* = 0.536]. Thus, priming of responses was significant only for High-M squiggles in Experiment 1. Furthermore, analysis of the influence of the study-test color change indicated selective influence of color change on priming for High-M squiggles. In Experiment 1, RTs for High-M squiggles were significantly faster for the same-colored old items compared to different-colored old items [*t*_(13)_ = −2.434, *p* < 0.05]. In contrast, there was no significant difference for Low-M squiggles in Experiment 2 [*t*_(13)_ = −0.672, *p* = 0.513] (Table 1).

**Table 1:** **Mean RT(ms) and standard error of meaningfulness ratings during the Day 2 test**.

Experiment 1 (High-M)		Experiment 2 (Low-M)	
Old (all)	797(31)***	Old (all)	757(20)
*Same-old*	785(33)***	*Same-old*	755(19)
*Different-old*	808(30)*	*Different-old*	760(22)
New	827(33)	New	762(24)

Numbers in parenthesis indicate standard error of the mean. Significant priming effects relative to a baseline from new items are indicated with: *p < 0.05, **p < 0.01, or ***p < 0.001.

### Experiment 3 and 4

#### Behavior

In Experiment 3, the probability that a squiggle was rated as High-M was 44.1% (SE = 3.1%) and Low-M was 56% (SE = 3.1%). In Experiment 4, the probability that a squiggle would be rated as High-M was 37.3% (SE = 1.8%) and Low-M was 61.9% (SE = 1.9%).

A 2 × 4 repeated measures ANOVA was run with squiggle repetition (old, new) and response type (remember, know, guess, new). Additional multiple comparisons between repetition conditions for each response type were conducted with Bonferroni correction for both Experiment 3 and 4. Experiment 3 exhibited a significant 2-way interaction [*F*_(1.367,17.769)_ = 39.393, *p* < 0.001]. The hit rate was significantly greater than the false alarm rate for the remember response (*p* < 0.001) and the know response (*p* = 0.001), suggesting that recognition on Day 2 was above chance levels. False alarms significantly outnumbered hits for the guess response (*p* < 0.001) and correct rejections significantly outnumbered misses for the new response (*p* = 0.001). Experiment 4 also exhibited a significant 2-way interaction (*p* < 0.001). The hit rate was significantly greater than the false alarm rate for the remember response (*p* < 0.001) and the know response (*p* < 0.001), again suggesting that recognition on Day 2 was above chance levels. False alarms significantly outnumbered hits for the guess response (*p* < 0.001) and correct rejections significantly outnumbered misses for the new response (*p* < 0.001). These results indicate successful recognition memory for Experiments 3 and 4.

**Figure 2:**
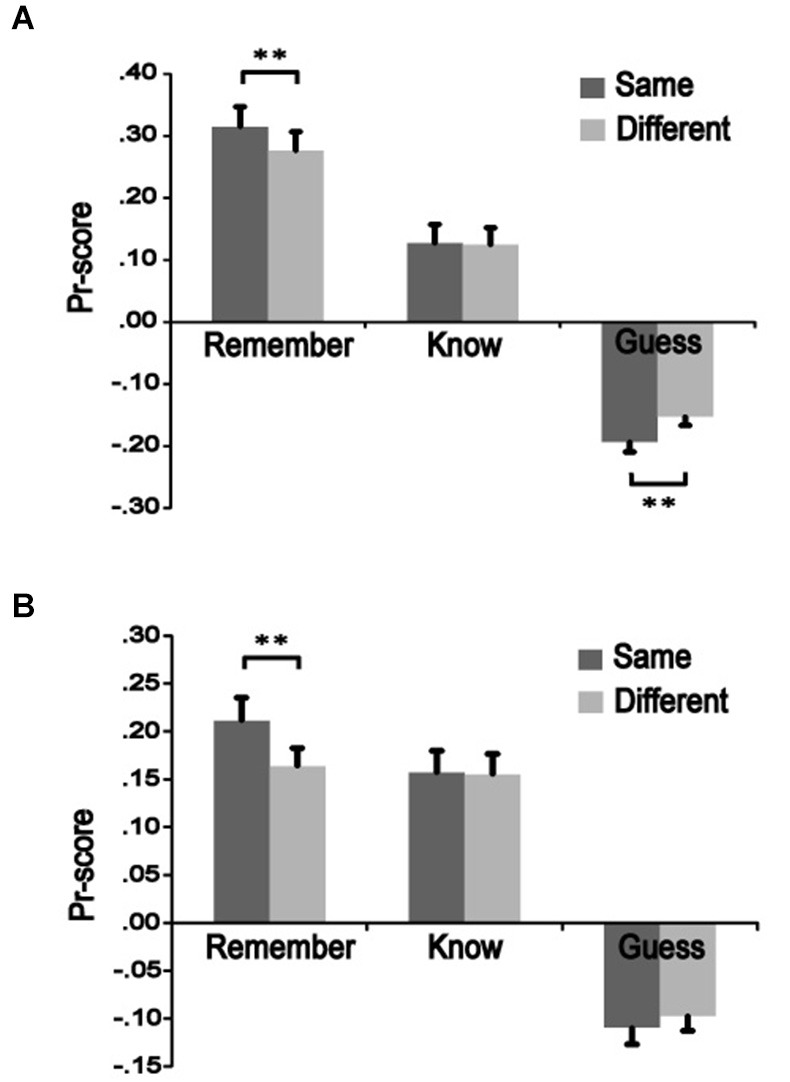
**The influence of color manipulation on recognition accuracy**. Pr-scores were calculated to determine effects of color change on recognition accuracy. Squiggles presented during a study phase were shown again during a test phase as either the same or a different color. Recognition precision of same-colored was compared to different-colored squiggles in the remember, know, and guess conditions. **(A)** Values for High-M items in Experiment 3. **(B)** Values for Low-M items in Experiment 4. ** *p* < 0.01.

To measure recognition accuracy, we used discrimination (Pr) scores, which are a normalized measure of the “hit rate to old items” minus the “false alarm rate to new items” (cf. Snodgrass and Corwin, 1988). Pr scores were significantly higher for the same-remember condition than the different-remember condition in both Experiments 3 [*t*_(13)_ = 3.846, *p* = 0.002] and 4 [*t*_(15)_ = 3.367, *p* = 0.004], indicating better remember performance when color was the same from study to test vs. when it changed. The same-know condition did not differ significantly from the different-know for either Experiment 3 [*t*_(13)_ = 0.335, *p* = 0.743] or 4 [*t*_(15)_ = 0.171, *p* = 0.867]. The different-guess condition was significantly greater that the same-guess in Experiment 3 [*t*_(13)_ = 3.891, *p* = 0.002] but not in Experiment 4 [*t*_(15)_ = 1.149, *p* = 0.269] (Figure 2). These results indicate that color change had an influence on remember responses but no effect on know responses, suggesting different effects on recollection vs. familiarity-based responses.

#### ERP correlates of color-change for meaningful and meaningless stimuli

**Figure 3:**
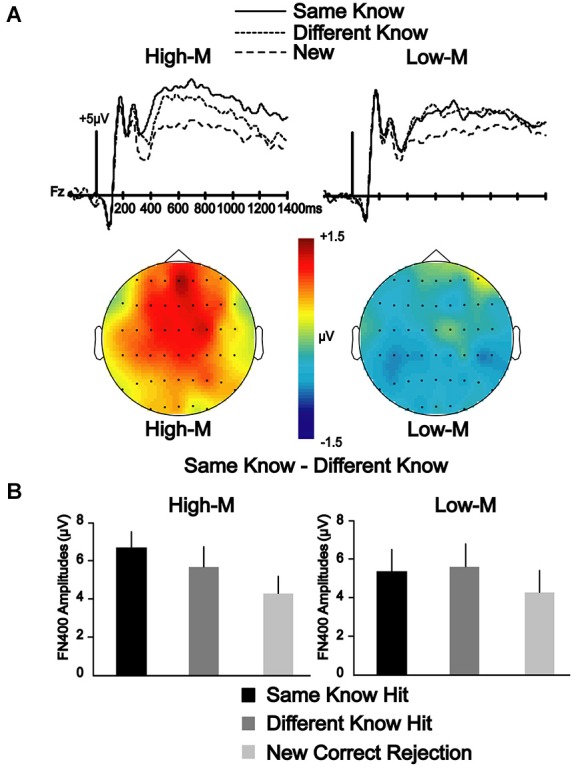
**ERP correlates of color manipulation on FN400 for High-M and Low-M stimuli endorsed with know responses. (A)** ERPs indicate the effect of color manipulation on amplitude of FN400 that occurred for know responses in Experiment 3 (High-M stimuli), but was not evident in Experiment 4 (Low-M). Topographic maps of the amplitude difference between Same-Know and Different-Know conditions shows a clear FN400 effect for High-M that is lacking for Low-M. **(B)** Mean amplitude of FN400 potentials corresponding to these effects for 350–500 ms for the frontal electrode cluster.

A 2 × 3 repeated measures ANOVA was conducted between two conditions (same-know, different-know) for three electrode clusters (frontal, central, parietal) at 350–500 and 500–700 ms latency intervals to determine if the differences in neural correlates of color effects depended on whether the stimuli were meaningful or meaningless. In Experiment 3, the main effect of condition was significant [*F*_(1,13)_ = 5.084, *p* < 0.05] at 350–500 ms, suggesting that the amplitudes for the same-know condition was significantly more positive than the different-know condition. The condition-by-cluster interaction was not significant [*F*_(2,26)_ = 0.534, *p* = 0.593], suggesting a similar effect for all clusters. In Experiment 4, the main effect of the condition was not significant [*F*_(1,14)_ = 0.684, *p* = 0.422] at 350–500 ms. Condition-by-cluster interaction was also not significant [*F*_(1.338,18.738)_ = 0.375, *p* = 0.609]. These results suggest that FN400 amplitudes varied due to color change, but only for meaningful stimuli in Experiment 3 (Figure 3).

A similar analysis was performed for know responses at the 500–700 ms (LPC) latency interval. The main effect of condition was not significant in Experiment 3 [*F*_(1,13)_ = 1.910, *p* = 0.190] or 4 [*F*_(1,14)_ = 0.956, *p* = 0.345]. The condition-by-cluster interaction was also not significant for Experiment 3 [*F*_(2,26)_ = 0.133, *p* = 0.876] or 4 [*F*_(1.080,15.118)_ = 0.086, *p* = 0.792]. Color change thus had no influence on know ERPs during the LPC interval for either meaningful or meaningless stimuli in Experiments 3 and 4.

**Figure 4:**
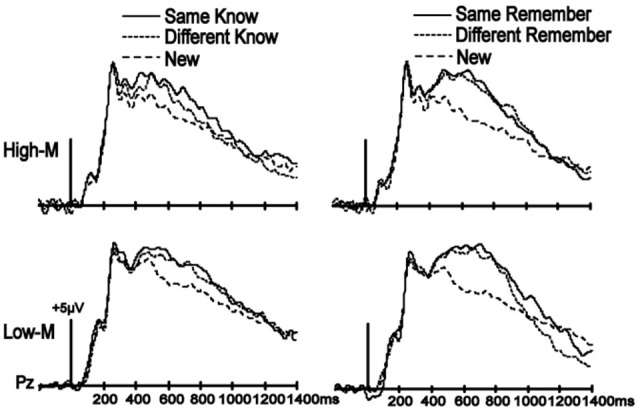
**No influence of color manipulation on LPC**. ERPs are shown averaged for the parietal electrode cluster used to assess LPC. Despite clear LPC enhancements for all old items relative to new items for both High-M (Experiment 3) and Low-M (Experiment 4) squiggles, there were no effects of color manipulation (Same-Know vs. Different-Know, and Same-Remember vs. Different-Remember).

A similar analysis was used to identify effects on ERP correlates of recollection for both latency intervals to determine color change effects on neural correlates of recollection. No main effects of condition or condition-by-cluster interactions were significant for either Experiment 3 or 4, as all *p*-values were > 0.1 (Figure 4). These results indicate that color change does not influence ERP correlates of remember responses for either High-M or Low-M stimuli.

#### ERP correlates of recognition

**Figure 5:**
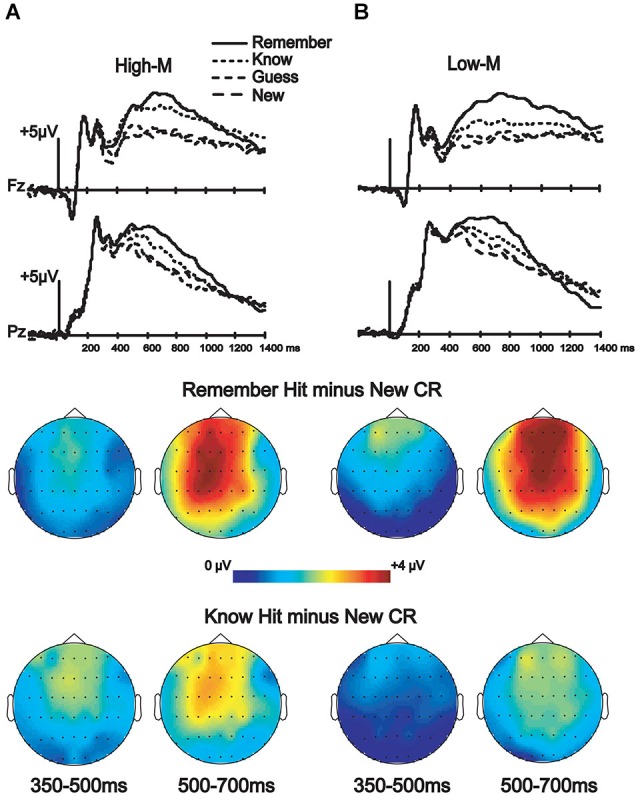
**ERP correlates of recognition for Experiments 3 (High-M) and 4 (Low-M)**. ERPs and topographic effects corresponding to the recognition memory categories (new, old guess, old know, and old remember) and topographic plots of difference amplitudes showing old/new effects for remember and know items at two indicated latency intervals. **(A)** High-M squiggle (Experiment 3) ERPs are shown on the left. **(B)** Low-M squiggle (Experiment 4) ERPs are shown on the right.

ERPs from correctly categorized old items (remember and know responses) were analyzed against ERPs of correctly categorized new items (new responses) in order to explore the neural correlates of recognition performance for High-M and Low-M squiggles, irrespective of color change (Figure 5). This analysis was conducted in order to validate our general patterns of ERPs with respect to previous findings using similar stimuli. The ANOVA results are summarized in Table 2. These results indicate reliable old/new clusters at all clusters, with greater amplitude effects for remember than know responses.

**Table 2:** **Summary of the conducted repeated measures ANOVA on ERPs data for ERP correlates of recognition**.

	Experiment 3 (High-M)		Experiment 4(Low-M)	
	Condition-by-Cluster Interaction	Pairwise Comparisons	Condition-by-Cluster Interaction	Pairwise Comparisons
	350–500 ms *F*_(4,52)_ = 3.83*** $\eta_{p}^{2}$ = 0.23 500–700 ms *F*_(2.03,26.4)_ = 3.30* $\eta_{p}^{2}$ = 0.20	Comparing Remember, Know, and New conditions	350–500 ms *F*_(4,56)_ = 12.75*** $\eta_{p}^{2}$ = 0.48 500–700 ms *F*_(2.29,32.03)_ = 4.50*** $\eta_{p}^{2}$ = 0.24	Comparing Remember, Know, and New conditions
**350–500 ms** *Frontal*	Condition Main effect *F*_(2,26)_ = 16.53*** $\eta_{p}^{2}$ = 0.56	Remember > New *F*_(1,13)_ = 20.01*** $\eta_{p}^{2}$ = 0.61 Know > New *F*_(1,13)_ = 34.04*** $\eta_{p}^{2}$ = 0.72	Condition Main effect *F*_(2,28)_ = 13.98*** $\eta_{p}^{2}$ = 0.50	Remember > New *F*_(1,14)_ = 22.58*** $\eta_{p}^{2}$ = 0.62 Know > New *F*_(1,14)_ = 13.51*** $\eta_{p}^{2}$ = 0.49 Remember > Know *F*_(1,14)_ = 4.60* $\eta_{p}^{2}$ = 0.25
**350–500 ms** *Central*	Condition Main effect *F*_(2,26)_ = 15.44*** $\eta_{p}^{2}$ = 0.54	Remember > New *F*_(1,13)_ = 16.56*** $\eta_{p}^{2}$ = 0.56 Know > New *F*_(1,13)_ = 21.96*** $\eta_{p}^{2}$ = 0.63	Condition Main effect *F*_(2,28)_ = 5.89*** $\eta_{p}^{2}$ = 0.30	Remember > New *F*_(1,14)_ = 11.01*** $\eta_{p}^{2}$ = 0.44
**350–500 ms** *Parietal*	Condition Main effect *F*_(2,26)_ = 5.50** $\eta_{p}^{2}$ = 0.30	Remember > New *F*_(1,13)_ = 9.29*** $\eta_{p}^{2}$ = 0.42 Know > New *F*_(1,13)_ = 5.46** $\eta_{p}^{2}$ = 0.30	Condition Main effect *F*_(2,28)_ = 2.19 $\eta_{p}^{2}$ = 0.14
**500–700 ms** *Frontal*	Condition Main effect *F*_(2,26)_ = 46.59*** $\eta_{p}^{2}$ = 0.78	Remember > New *F*_(1,13)_ = 69.79*** $\eta_{p}^{2}$ = 0.84 Know > New *F*_(1,13)_ = 59.97*** $\eta_{p}^{2}$ = 0.82 Remember > Know *F*_(1,13)_ = 6.89** $\eta_{p}^{2}$ = 0.35	Condition Main effect *F*_(2,28)_ = 40.34*** $\eta_{p}^{2}$ = 0.74	Remember > New *F*_(1,14)_ = 68.26*** $\eta_{p}^{2}$ = 0.83 Know > New *F*_(1,14)_ = 22.19*** $\eta_{p}^{2}$ = 0.61 Remember > Know *F*_(1,14)_ = 22.32*** $\eta_{p}^{2}$ = 0.61
**500–700 ms** *Central*	Condition Main effect *F*_(2,26)_ = 30.75*** $\eta_{p}^{2}$ = 0.70	Remember > New *F*_(1,13)_ = 44.02*** $\eta_{p}^{2}$ = 0.77 Know > New *F*_(1,13)_ = 24.16*** $\eta_{p}^{2}$ = 0.65 Remember > Know *F*_(1,13)_ = 9.46*** $\eta_{p}^{2}$ = 0.42	Condition Main effect *F*_(2,28)_ = 36.268*** $\eta_{p}^{2}$ = 0.72	Remember > New *F*_(1,14)_ = 67.45*** $\eta_{p}^{2}$ = 0.83 Know > New *F*_(1,14)_ = 18.50*** $\eta_{p}^{2}$ = 0.57 Remember > Know *F*_(1,14)_ = 19.38*** $\eta_{p}^{2}$ = 0.58
**500–700 ms** *Parietal*	Condition Main effect *F*_(2,26)_ = 13.78*** $\eta_{p}^{2}$ = 0.52	Remember > New *F*_(1,13)_ = 23.62*** $\eta_{p}^{2}$ = 0.65 Know > New *F*_(1,13)_ = 7.85** $\eta_{p}^{2}$ = 0.38 Remember > Know *F*_(1,13)_ = 7.33** $\eta_{p}^{2}$ = 0.36	Condition Main effect *F*_(2,28)_ = 26.04*** $\eta_{p}^{2}$ = 0.65	Remember > New *F*_(1,14)_ = 46.68*** $\eta_{p}^{2}$ = 0.77 Know > New *F*_(1,14)_ = 19.71*** $\eta_{p}^{2}$ = 0.59 Remember > Know *F*_(1,14)_ = 9.23*** $\eta_{p}^{2}$ = 0.40

Insignificant statistical data are not presented in the table. The Symbol “>” shown in the phrase, for example, “Remember > New” in pairwise comparisons column means “amplitudes in the remember condition are significantly more positive than those in the new condition. * p < 0.06 ** p < 0.05 *** p < 0.01. $\eta_{p}^{2}$ (partial eta squared) was used as index of effect size.

## General discussion

The purpose of these experiments was to identify distinct effects of a perceptual manipulation (color change) on behavior and neural correlates of perceptual and conceptual processing during recognition memory. We first showed that color change disrupted conceptual priming, but only for squiggle stimuli that were perceived as meaningful (High-M), not for those perceived as relatively meaningless (Low-M). This demonstrates that color change can alter conceptual processing, but only when the stimulus is relatively meaningful and therefore is capable of supporting conceptual processing. Next, we investigated the effects of the color change manipulation on ERP correlates of recognition memory, reasoning that ERP correlates of recognition altered by the color change manipulation would reflect conceptual processing during recognition memory. Color change significantly influenced FN400 correlates of recognition for meaningful High-M squiggles, but not for Low-M squiggles. Taken together, these findings indicate that FN400 effects reflected conceptual processing during a recognition test, and that the effects of color change manipulations on FN400 reflected a perceptual manipulation (color) having an influence on conceptual processing.

The variability of meaningfulness ratings in all four experiments reliably justifies the individual rating method we used for each subject. On the basis of these methods, our behavioral studies in Experiment 1 and 2 found that conceptual priming was only evident for stimuli rated by individual subjects as meaningful, which was in line with prior studies using various stimuli, for example, squiggles (Voss and Paller, 2007; Voss et al., 2010b, 2012a), obscure words (Voss et al., 2010a), and ancient Chinese characters (Hou et al., 2013). On the basis of prior studies, we further found color effects on response facilitation are selective for High-M stimuli. These results taken together demonstrate that meaningfulness plays a key role in the color effects on conceptual priming when all other conditions are held constant.

ERP correlates of familiarity have been debated in recent years. The mid-frontal old/new effect has most frequently been regarded as the neural indicator of familiarity (reviewed in Mecklinger, 2006; Rugg and Curran, 2007). However, some experiments have shown that familiarity occurs even without FN400 effects (Yovel and Paller, 2004; Danker et al., 2008; Voss and Paller, 2009) suggesting that perhaps FN400 is not a unique indicator of familiarity. Other findings have indicated that FN400 effects could reflect conceptual processing during recognition (Olichney et al., 2000; Voss and Paller, 2006, 2007; Voss et al., 2010a; Voss and Federmeier, 2011; Hou et al., 2013). For instance, Voss et al. (2010a) used obscure words as stimuli so as to dissociate the neural correlates of familiarity and conceptual processing; the results indicated that FN400 effects were found only for the words that elicited meaningful associations, despite matched familiarity with meaningless words that did not produce FN400 effects. In contrast, familiarity for both meaningful and meaningless words was associated with similar late-positive ERP repetition effects. If FN400 effects indeed reflect conceptual processing, then they could be identified with respect to familiarity in many circumstances because familiarity can often be based on attributions of fluency of conceptual processing (Jacoby and Whitehouse, 1989; Whittlesea, 1993). In other words, FN400 could reflect an influence of conceptual processing on familiarity when meaningful items are used because conceptual fluency contributes to familiarity in some (but not all) circumstances (Lucas et al., 2012; Voss et al., 2012b).

The old/new effects that we describe at 350–500 ms are highly similar to FN400 effects described in previous studies. We found that the color change manipulation influenced behavioral indicators of conceptual priming only for High-M squiggles, suggesting that conceptual processing occurring selectively for High-M squiggles can be facilitated when color is consistent from study to test, and is disrupted by color change. Importantly, the ERP correlates during recognition of the color-change manipulation were selective for the FN400 and for High-M squiggles. This indicates that FN400 ERPs reflected conceptual priming during recognition that occurred selectively for High-M squiggles. In a previous study, a similar color change manipulation was used with squiggles and effects were also identified on FN400 potentials (Groh-Bordin et al., 2006). However, in that study, squiggle meaningfulness was not characterized. It is therefore likely that the effects of color change on FN400 were due to the subset of squiggles in that experiment that subjects found to be relatively high in meaningfulness, and that they reflected conceptual priming rather than any perceptual influence on the FN400 *per se*. In general, it is problematic to manipulate the physical attributes of a stimulus and assume that stimulus meaningfulness will remain constant. This same issue exists for other ERP studies that have noted influences of perceptual changes on FN400 effects, in that they also did not ascertain the effects of these manipulations on either behavioral or neural indicators of conceptual processing (Schloerscheidt and Rugg, 2004; Groh-Bordin et al., 2006; Ecker et al., 2007a,b; Curran and Doyle, 2011).

The LPC old/new effects that we identified were consistent with these brain potentials as correlates of remember and know responses (i.e., reflecting both self-reported recollection and familiarity). LPC amplitudes were greatest for recollection, less for familiarity, and least for correctly identified new items, and mostly independent of stimulus meaningfulness and color change. This is in accordance with previous findings showing that LPC amplitude tracks increasing confidence of recognition without significant variation based on meaningfulness (see Voss and Paller, 2009).

One divergence between our study and previous studies that have used minimally meaningful stimuli, including the Low-M squiggles used here, is that no reliable FN400 effects were identified in those previous studies (Voss and Paller, 2007; Voss et al., 2010a), whereas the Low-M condition occur with a reliable FN400 effect in our study, albeit with a smaller amplitude than for the High-M condition (Figure 5). It is possible that our use of exclusively Low-M stimuli in Experiment 4 promoted distinctiveness of individual Low-M items, whereas those previous studies used mixtures of High-M and Low-M stimuli in each test block. Increased distinctiveness could lead to identification of meaningful features in a greater number of Low-M items. It is thus important to emphasize that the Low-M condition is not necessarily entirely without conceptual processing, and the specific methods used here could have emphasized this processing slightly with respect to previous studies with mixed meaningfulness in each test. Nonetheless, the FN400 amplitude for know responses was greater for High-M compared to Low-M stimuli, thus indicating that it was related to conceptual processing associated with meaningfulness. Indeed, recognition performance was matched for High-M and Low-M know responses, indicating that gross differences in memory accuracy or strength were unlikely to have produced this difference. Taken together with the selective effects of color change on FN400 correlates of know responses for High-M items and on behavioral measures of conceptual priming for High-M stimuli, this finding underscores the relationship between FN400 recognition effects and conceptual processing.

Finally, our results help clarify the nature of the distinction between perceptual and conceptual processing. Although these are often considered distinct entities that can be easily separated, for instance in tests of perceptual vs. conceptual priming (e.g., Jacoby, 1983; Graf and Mandler, 1984; Smith and Branscombe, 1988; Blaxton, 1989; Graf and Ryan, 1990; Srinivas and Roediger, 1990; Rappold and Hashtroudi, 1991; Weldon, 1991, 1993; Cabeza and Ohta, 1993; Challis et al., 1993; Weldon et al., 1995; Carlesimo et al., 1996), we found that the influence of what would normally be considered a purely perceptual manipulation can actually have a profound effect on conceptual processing, both in priming behavior and in ERP correlates of recognition memory. This finding has implications for studies that pit perceptual vs. conceptual manipulations against each other in order to identify cognitive and neural processing relevant for perception and memory (Clarke and Morton, 1983; Masson, 1986; Jacoby and Hayman, 1987; Groh-Bordin et al., 2006; Nyhus and Curran, 2009; Herzmann et al., 2012). Furthermore, these findings help clarify when simple perceptual features will influence the meaning of a stimulus, and the ramifications of this influence on meaning for stimulus processing and memory.

## Conflict of interest statement

The authors declare that the research was conducted in the absence of any commercial or financial relationships that could be construed as a potential conflict of interest.
